# Ultrasonic Dispersion for Iron Recovery from Slime Tailings: Microprocesses Unveiled through Molecular Dynamics Simulations

**DOI:** 10.1021/acs.langmuir.4c03676

**Published:** 2025-03-17

**Authors:** Lucas Andrade Silva, Letícia Maia Prates, Alexandre Moni Pereira, Julio Cesar Guedes Correia, Michelle Lacerda Sales Marques, Inna V. Filippova, Lev O. Filippov

**Affiliations:** † Ministry of Science, Technology and Innovation (MCTI), Molecular Modeling Laboratory, Centre for Mineral Technology (CETEM), Av. Pedro Calmon, 900, Ilha da Cidade Universitária, Rio de Janeiro, RJ 21941-908, Brazil; ‡ Iron Ore Beneficiation Development Team, Vale S/A, Belo Horizonte, Minas Gerais 34006-270, Brazil; § CNRS, GeoRessources, Université de Lorraine, F54000 Nancy, France

## Abstract

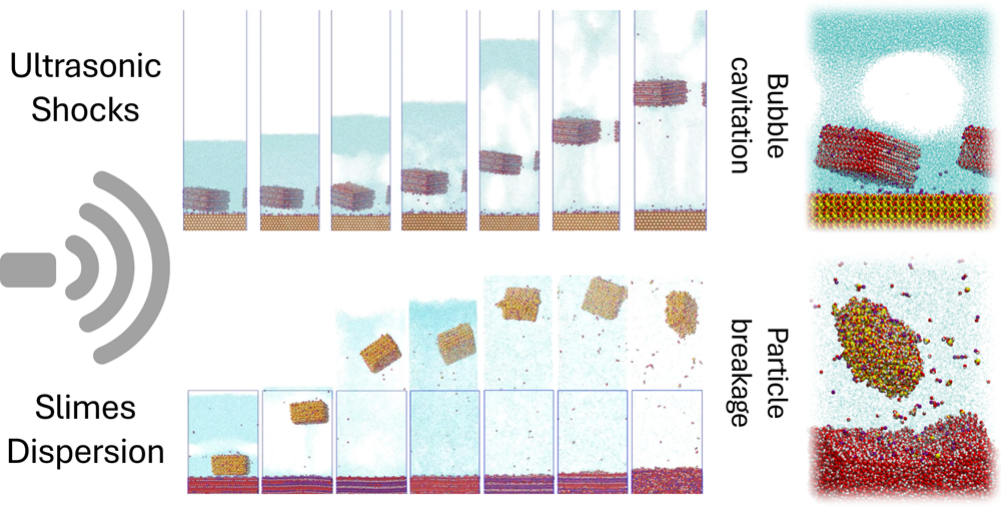

Chemical dispersion has been commonly used to mitigate the negative effects of ultrafine particles in iron ore concentration processes. However, mechanical solutions such as ultrasound are proving to be more effective and without harmful side effects. This study compared the performance of different dispersants and ultrasound as pretreatments for reverse cationic flotation of goethite-rich slime tailings through sedimentation, dispersion, and flotation tests, along with particle size analysis. Additionally, large-scale molecular dynamics simulations were used for the first time to investigate the effects of ultrasonic shockwaves on mineral particle interactions. The results showed that ultrasonication is a superior pretreatment, enhancing particle dispersion and separation performance, cleaning mineral surfaces, and improving flotation results. Ultrasound achieved an increase in metallurgical recovery of around 9% while using only a dispersant reagent did not reach 5%. Simulations demonstrated the known effects of ultrasound, such as extreme temperature, bubble cavitation, and particle detachment, revealing the crucial microscopic mechanisms involved in particle separation by sonic waves. This study bridges experimental data with computational simulations, offering a comprehensive understanding of ultrasonication’s effects on particle separation, paving the way for more efficient and sustainable processing technologies.

## Introduction

Goethite (FeO(OH)) is one of the major iron minerals in the Earth’s crust and is often present in fine particles (below 20 μm). Most commercial iron ores are mainly composed of hematite and magnetite. However, the depletion of high-grade reserves has made hydrated minerals, such as goethite, an increasingly important future source of iron.^1^ This is particularly critical in the context of raw materials for steel and alloy production across various sectors, including construction and manufacturing. The main gangue mineral in commercial iron ores is quartz, but fine iron ores often contain other deleterious components, such as alumina, which originates from ultrafine clay minerals, such as kaolinite and gibbsite. This compositional diversity makes the recovery of precious minerals challenging. The small mass and high specific surface area of fine particles result in low collision and adhesion to bubbles during the flotation concentration process, leading to significant losses due to entrainment.^1,2^ As such, under the current industrial standard, most hydrated and fine/ultrafine mineral particles are disposed of in tailing dams after size classification and desliming operations to ensure that concentration processes meet the required final product grade.^3^ Over the decades, this practice has created significant environmental liability.^4^ However, it also holds the potential to supply industry by reclaiming this source of iron, requiring scientific and technological advancements to enable its sustainable and economically viable recovery.

A common phenomenon in mineral processing pulps containing ultrafine particles is slime coating. In fine iron ore, slime coating refers to the adhesion or heteroaggregation of ultrafine particles and impurities onto the surface of larger particles. This phenomenon occurs in the beneficiation of various ores, including sulfides (e.g., sphalerite, galena), oxides (e.g., hematite, wolframite), salt minerals (e.g., fluorite, potash), as well as in coal and bitumen processing.^5,6^ This interfacial phenomenon is primarily driven by electrostatic attraction and poses a significant challenge in separation processes such as flotation and magnetic separation,^1,5−7^ because its presence alters the surface properties of the ore as well as keeping the particles to be separated tightly bound.^1,8^ This results in lower-grade concentrates, increased chemical consumption, and extended processing times, ultimately driving up operational costs and the loss of valuable materials as waste.

To mitigate slime coatings, increasing the zeta potential of the particles can enhance surface charge magnitude, promoting greater electrostatic repulsion and improving dispersion. Adjusting the pulp pH, often by adding alkalis such as NaOH, can sometimes help stabilize the system. However, the application of dispersant reagents such as hexametaphosphate (SHMP) and sodium silicate (SS) can be more effective and necessary in some cases.^8^ The application of these chemicals for iron ore slimes remains elusive since contradictory results have been presented in previous studies, some of them reporting the dispersants acting as quartz depressants or competing and hindering the action of the collector or depressant reagents used subsequently.^9−11^ Employing advanced surface chemistry techniques and optimizing chemical reagent strategies can significantly enhance the separation efficiency and improve the quality of the final product.

Sredić and Tankosić^8^ studied the effect of pH and different dispersants on the surface charge of a goethite sample. The highest zeta potential value was achieved with the application of SHMP. Once the pulp is dispersed, ultrafine removal through desliming may be sufficient to ensure good results in subsequent concentration steps. On the other hand, this stage of the process can also lead to significant iron losses. A contrasting approach to efficient dispersion is the use of mechanical means instead of chemical means. For example, high-intensity conditioning (HIC)^12^ is based on the application of high shear rates by using a more selected hydrodynamic setup in the pulp mixing and increased RPM, allowing more turbulent conditions that transfer more energy into the pulp.

Another increasingly popular method for particle dispersion involves the application of mechanical force through ultrasound waves.^13,14^ Ultrasonication (US) has emerged as an effective method for improving particle dispersion as flotation pretreatment in mineral processing and resource recycling.^14−20^ Operating within the frequency range of 20–50 kHz, ultrasound induces transient cavitation, where microbubbles rapidly form and collapse on particle surfaces. This process generates extreme local temperature (∼5000 K) and pressure (∼50,000 bar) gradients and high-speed jet flows which facilitate surface cleaning by removing slime coatings and exposing fresh surfaces for reagent adsorption.^21^ Additionally, ultrasonication promotes sonochemical effects, including free radical formation, chemical bond breakage, and surface structure disruption.^1,5,14,20,22^ These effects enhance reagent dispersion, improve mass transfer, and generate micro- and nanobubbles that increase the flotation efficiency. Ultrasonication is particularly effective in breaking down fine particle agglomerates and cleaning mineral surfaces, making it a highly efficient energy input method for colloidal particle dispersion. In recent work, Batista e Silva et al.^19^ tested ultrasonication and chemical dispersion together for the reverse flotation of kaolinite from bauxite, diminishing slime coating and achieving better separation response with ultrasound. They highlighted that previous works investigating the synergic effect of ultrasound and chemical dispersion were not found in the literature. We also aim to tackle this gap in this study.

In addition to classical experimental approaches, computer simulations play a crucial role in elucidating the underlying mechanisms of particle dispersion and separation processes, such as flotation, at a molecular level.^23,24^ Over the past two decades, numerous studies have employed molecular simulations and quantum chemical calculations to investigate adsorption mechanisms and guide reagent selection and design.^23,25^ Recent studies have used molecular dynamics simulations to explore the effects of environmental effects, such as pH and concentration, as well as surface chemistry and functionalization on the interaction between particles, for example,^7,26,27^ going beyond classical colloidal interaction theories. Furthermore, in the context of ultrasonication, simulations enable detailed observation of cavitation effects, the role of shockwaves on particle surfaces, and the resultant structural changes at a nanoscopic scale.^28,29^ Thus, integrating computational simulations with experimental methods enhances the overall understanding of mechanisms and separation efficiency.^23^

In our previous studies, different strategies were tested to enhance the separation efficiency of goethite-rich slime tailings. In the flotation reagent sphere, a new amidoamine molecule has been shown to be more selective than conventional etheramines, even without a depressant.^30−33^ More recently, combinations of this amidoamine with frothers were tested and compared to a ternary amidoamine-etheramine-MIBC formulation, which was comprehensively characterized and tested against real slime samples, single minerals, and artificial mixtures. A multiscale approach was employed across several studies to understand its molecular mechanisms and optimize operational conditions.^18,33,34^ This new reagent formulation presents a promising alternative for these difficult-to-process ores and tailings.

In our previous investigation, ultrasound parameters were evaluated as pretreatment for the flotation separation of the same goethitic slime sample as in this study.^18^ A brief comparison of ultrasound with dispersion using only NaOH revealed that dispersion at pH 10.5 resulted in slightly inferior flotation performance compared to no dispersion at all. Dispersion at natural pH under sonication led to even worse results, likely due to the zeta potential of silicates and iron oxides, combined with the enhanced mixing caused by ultrasound, which promoted particle heteroaggregation. However, at pH = 10.5 with ultrasound, the separation efficiency improved significantly. Particle size distribution analysis indicated an increase in the population of both finer and coarser particles compared with the sample before dispersion tests. This suggests that ultrasound facilitated both slime coating removal and aggregation of another particle fraction. Given the good flotation selectivity observed, this aggregation was attributed to the possible homoaggregation of iron minerals.

Building on our previous research, this study evaluates the effectiveness of mechanical dispersion via sonication versus chemical dispersion using different reagents in the pretreatment of goethite-rich ultrafine iron ore slimes prior to reverse cationic flotation. Given the challenges and importance of ultrafine tailing recovery, we explore the potential novelty of substituting or combining chemical dispersants with ultrasound to enhance the process. By combining experimental approaches with molecular modeling, we aim to elucidate the underlying mechanisms of ultrasound-assisted dispersion, an approach not yet adopted in the mineral industry. Understanding these phenomena can provide insights into sustainable process intensification and facilitate future scale-up for enhanced metallurgical recovery from tailings. The paper is organized as follows. First, we present the samples, chemicals, and methods used in experimental tests, where two types of dispersants were tested and compared with and without ultrasound application. Second, molecular modeling techniques were used to obtain a molecular picture as a proof-of-concept to understand the effects caused by ultrasound and how they can affect the dispersion and separation processes. We present an unprecedented computational methodology for studying the effects of ultrasonic shockwaves on the interaction between a probe nanoparticle and a mineral surface. This is the first instance, to the best of our knowledge, where molecular simulations of sonic shockwaves on mineral colloidal systems have been presented in the literature. Finally, we discuss the observed results, the mechanisms, and implications.

## Methodology

### Experimental Details

#### Sample Characterization

An ultrafine tailings sample with goethitic characteristics from the Iron Quadrangle, in Brazil, was collected from a beneficiation plant during a stable production period. The sample was homogenized and divided using a splitter to obtain subsamples for characterization and laboratory tests. To obtain the particle size distribution, the samples were submitted to a laser light scattering method with a helium–neon laser optical system Mastersizer 3000 (Malvern Panalytical, Malvern, U.K.). The equipment performs three measurements of the size distribution automatically, at 10 s intervals, with the result being the average. It then calculates the result of the granulometric distribution through internal mathematical models. Chemical analysis via X-ray fluorescence was performed and the sample has Fe, SiO_2_, Al_2_O_3_, and LOI content of 51, 12.9, 4.1, and 8.5%, respectively. The main iron minerals are goethite (64%) and hematite (19.5%), and the main gangue minerals are quartz (11%) and kaolinite (4%). The minerals were identified by X-ray diffraction and QEMSCAN (*quantitative evaluation of minerals by scanning electron microscopy)*. The particle size distribution by laser diffraction indicated that the sample has 95% of the material less than 45 μm and 63% less than 10 μm. More detailed information can be found in our previous work.^18^

#### Reagents

Chemical and physical dispersion were evaluated. The selected reagents were sodium hexametaphosphate (SHMP), which is widespread in the literature,^1^ and sodium polycarboxylate, commercially known as Solutrix D (SD). For physical dispersion, ultrasound was applied. For the flotation steps, the recently introduced collector-frother reagent Flotinor 16939 was selected, as it has been proven to be suitable for goethitic ore reverse flotation without the need for a depressant.^18,33^ This reagent is a ternary mixture of etheramine, amidoamine, and an alcoholic frother and has been the subject of recent studies.^18,33,34^ The reagents used are given in Table 1.

**Table 1:** Reagents Used in the Tests

name	role	chemical formula	provider
sodium hexametaphosphate (SHMP)	dispersant	(NaPO_3_)_6_	Alfa Aesar
solutrix D (SD)	dispersant	(C_3_H_3_NaO_2_)_*n*_	Basf
sodium hydroxide	dispersant/pH modifier	NaOH	VWR Chemicals
Flotinor 16939	collector/frother	-	Clariant

#### Dispersion Tests

For chemical dispersion, different dosages were evaluated with and without simultaneous ultrasound treatment. An ultrasonic generator Sonoplus HD 3100 (Bandelin, Germany) with a rod waveguide (diameter 13 mm) and a maximum vibration amplitude of 97 μm with integrated amplitude control of 10–100% at a constant resonance frequency of 20 kHz providing an adjustable power range of 10–75 W was used as the ultrasound source. The dispersion was conducted in a flotation cell for 10 min residence time and pH adjusted to 10.5. The impeller rotation speed applied was 2980 rpm. Ultrasound intensity of 25% and residence time were selected based on the best flotation conditions in our previous investigation.^18^

#### Flotation Tests

Flotation tests were performed in a 400 mL cell (self-aerated MINEMET flotation machine) with a rotation speed of 1780 rpm. The dry basis mass of ore used was 32.5 g at a solids content of 10%. The collector conditioning time lasted for 1 min, and the flotation test duration was 4 min. All reagent solutions were prepared with 1% concentration. The first set of tests was performed to compare the dispersion prior to flotation using a dosage of a collector of 300 g/t at pH 10.5. The test parameters are described in Table 2. The flotation parameters were selected based on optimum conditions from our previous works.^18,32^ Flotation results from our previous work,^18^ under similar conditions but at varying US intensity in pretreatment were recalled for comparison.

**Table 2:** Test Parameters for the Flotation Studies with Different Dispersants

stage	dispersant	dispersant dosage (g/t)	collector dosage (g/t)	solids (%)	pH	US intensity (%)	US time (min)
1	NaOH	—	300	10	10.5	0, 25	10
2	SHMP	250, 500, 750	300	10	10.5	0, 25	10
3	SD	500, 750, 1500	300	10	10.5	0, 25	10

#### Sedimentation Tests

The study investigated how ultrasound treatment affects particle dispersion by using sedimentation tests to monitor sedimentation rates via the clear liquid–solid interface. Various ultrasound amplitudes (10, 25, 50, 75, and 100%) for 10 min and durations of 5, 10, and 15 min at 25% amplitude were tested, alongside at natural pH and pH 10.5. Pulp was initially stirred for 10 min at 500 rpm, adjusted to pH 10.5, treated with ultrasound, for each ultrasound amplitude and sent to laser size analysis. The test parameters are described in Table 3. To compare particle size distributions during sedimentation, samples from systems with dispersants and under ultrasound treatment at 25% amplitude were extracted using 5 mL pipettes at consistent depths of 3, 6, and 9 cm, after 0.5, 1.5, and 3 h.

**Table 3:** Sedimentation Tests Parameters

tests	dispersant	dispersant dosage (g/t)	solids (%)	pH
1	—	—	10	natural
2	NaOH	—	10	10.5
3	SHMP	500	10	10.5
4	SD	750	10	10.5

#### Computational Modeling

Molecular dynamics (MD) simulations using classical molecular mechanics force fields allow for the simulation of thousands of atoms for hundreds of nanoseconds. MD simulations are routinely used to study atomic-level interactions in detail and have become an effective tool for interpreting experimental results in various fields, including chemical and environmental engineering, mineral processing, and extractive metallurgy, all of which have seen significant growth.^23^ The effects of propagated shockwaves and sonication have been studied recently by molecular simulation approaches, focusing mostly on biological systems, such as cell membranes and amyloid fibrils.^28,29,35−37^ Depending on the frequency of the ultrasound used, the corresponding wavelength and characteristic period may not be computationally tractable. In such cases, simulations of single shockwave events can be representative.

A few studies in the literature have applied either a variable oscillating pressure approach^28,29,35,36^ or a single shockwave approach.^37−39^ The first entails the application of a barostat in which the pressure set point is defined by a time-dependent function, such as a sinusoidal, square, or triangular wave. In this way, the actual periodic behavior of the sound waves can be modeled. This method is especially suited for high-frequency waves so that the respective period can be observed. For example, a period of 1 ns corresponds to a high frequency of 1 GHz, and a low frequency of 20 kHz corresponds to a period of 50 μs. As such, to observe the complete period of a 20 kHz wave, a 50-μs-long simulation must be run (50 μs = 50,000 ns), which is unaffordable. In such cases, the use of a single shockwave approach is more attractive.

Some of the works considered the direct impact of the shockwave onto the systems (positive Δ*P*, compressing regime)^40^ while others considered the existence of nanobubbles previously generated by cavitation (generated in the rarefaction regime, negative Δ*P*) and the effect of the following positive pressure onto causing the bubble collapse.^37,39^ Additionally, another approach to mimic the effects of ultrasound is to simulate the extreme temperature and pressure that occur locally.^41,42^

Herein, to gain further insight into the microscopic mechanisms behind the effect of ultrasound on particle surfaces, we implemented a methodology based on large-scale atomistic molecular dynamics simulations. We employed an all-atom classical force field approach to include in detail all components without coarse-graining approximations. Similar methodologies have been used to study the effects of sonic shockwaves on biological systems,^28,29,35−37,40^ but this is the first time in the literature that such an approach has been applied to colloidal mineral systems. We applied a shockwave approach based on the collision of a rigid wall at varying velocities, mimicking the different energy inputs.

#### Molecular Models and Force Fields

Slime coating in iron ore processing can be modeled by considering an iron oxyhydroxide fine particle adhered to a silicate surface (herein called G-on-Q), or the other way around (Q-on-G). Herein the models were prepared considering goethite (G) and quartz (Q), in both possible configurations. The general conditions were considered as ambient temperature, 298 K, and pH ∼ 10, which is the usual pH used for dispersion and reverse flotation processes, as in the experimental section.

Surface models with dimensions of approximately 100 × 100 Å^2^ and particle models with dimensions of approximately 50 × 50 Å^2^ (projected area of 25 nm^2^), both with a thickness of ca. 3 nm, were prepared from our previously developed models.^31,43,44^ Particles were inserted above the surfaces, separated by 20 Å (counting from the surface topmost atoms to the particle lowermost atoms) alongside ca. 40,000 water molecules, the necessary amount of neutralizing counterions (ca. 160 Na^+^), and a region of vacuum, with a total box z length of 25 nm, using GEMS-Pack,^45^ PACKMOL,^46^ and GROMACS utilities^47^ and our own in-house scripts. The quartz on goethite and the goethite on quartz systems had 177,110 and 149,002 atoms, respectively.

The SPC/E^48^ and CLAYFF^49,50^ force fields (FF) were used to model the interatomic potentials. These models have been widely used for such kinds of water-mineral systems with success for the observation of many phenomena and prediction of several properties.^51^ The semi-ionic nature of ClayFF allows the simulated minerals to respond better to mechanical stress and strain and even break if forces are strong enough, which makes it more suitable than fully bonded force fields for studying the materials’ response to mechanical stress.^51^ It also enables the observation of breakage phenomena. All of these make this FF especially suitable for this study. A cutoff of 12 Å was set to calculate the short-range interactions, and the Particle Mesh Ewald (PME) method was employed to compute the long-range electrostatics. All MD simulations were carried out using GROMACS.^47^ LINCS algorithm was used to restrict the H-containing bonds.

#### Creation of the Slime Coating Models

**Figure 1:**
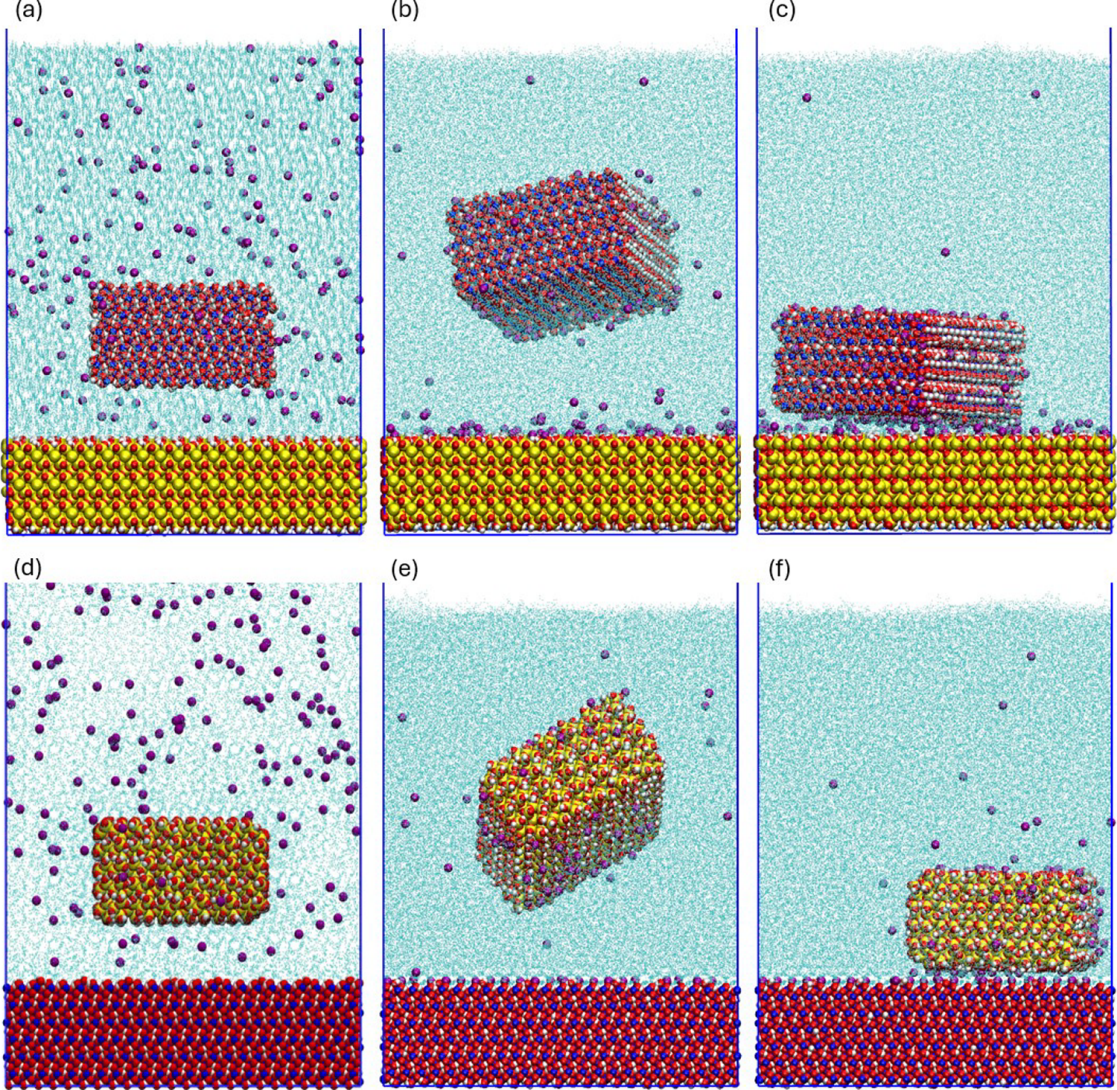
Molecular models for simulating slime coating. Top row: goethite particle onto quartz surface; bottom row: quartz particle onto goethite surface. (a,d) Initial models as-built; (b,e) snapshots after the initial equilibrium simulations after particle-surface repulsion; (c,f) snapshots of the slime coating condition created after SMD. Colors: cyan–water, red–oxygen, white–hydrogen, blue–iron, yellow–silicon, purple–sodium.

Starting from the configurations described above, equilibrium simulations were performed to allow the system to relax. The like-charged particle and surface repelled each other quickly, while the counterions rearranged around them, reaching a final, separated state, which remained stable for 80 ns simulation runs. After that, steered molecular dynamics simulations (SMD) were used to pull the particle close to the surface to create the slime coating condition. As they approach, the electrical double layers of both minerals are compressed together and the Na^+^ counterions are shared between them, establishing a cation bridging adhesion mechanism. This new state was highly stable throughout the 90 ns simulation runs. This is one of the possible mechanisms driving slime coating^5^ and was used as our model case. Figure 1 shows the models throughout the protocol steps.

#### Equilibrium MD Simulations

Additionally, to the equilibrium simulations carried out in the slime coating model creation, a few different conditions were assessed to probe the effect of thermodynamic variables on the probability of detaching the adhered goethite particle from the quartz surface. The v-rescale thermostat and the c-rescale semiisotropic barostat were used throughout unless otherwise noted. Conditions of high temperature and pressure were used to simulate the local effects caused by ultrasonication. The time step was set to 2 fs and a total simulation time of up to 65 ns.

#### Nonequilibrium MD Simulations

SMD was used to pull the particles from the surfaces into the solution. The corresponding force curves and work of adhesion were measured to probe the strength of the particle–particle interaction. The work of adhesion, *W*_a_, can be defined as the integral or the area under the curve of force versus distance. This approach is similar to that of a virtual atomic force microscopy (AFM) apparatus. A spring constant of 5000 kJ·mol^–1^·nm^–2^ was used to keep the stiff spring approximation valid, and a pull rate of 0.2 nm·ns^–1^ was used.

#### Shockwave Simulation Protocol

The pre-equilibrated slime coating models were subjected to collisions with a solid immovable wall boundary at varying initial velocities in the *z*-direction. This way, shockwave propagation was simulated. In this part, simulations were run under the microcanonical ensemble, NVE (constant number of particles, volume, and total energy).

Gromacs’ accelerate function was used to provide the surface model with an acceleration of 0.05 nm·ps^–2^ in the *z* direction, with 1 fs time step for 100 ps, to generate a trajectory of an accelerated system, in which each frame has a different, increasing, linear *z* velocity, *v_z_*. By extracting spaced frames, we obtain several starting configurations with different initial *z* velocities. These configurations were then recentered in the system and subjected to collision with an immovable wall boundary, using Gromacs’ walls function. By relative velocity and momentum conservation principles, this set up is equivalent to a piston wall moving toward the system at the same velocity. The boundary walls in both ends of the simulation box in the z direction were modeled by a Lennard-Jones potential using parameters of the silicon atom type in ClayFF, whose energy parameter is low enough to be considered as a rigid sphere. Box *z* length was adjusted to 80 nm, periodicity was kept only in the *xy* directions in these systems, and electrostatics were calculated by a modified 2D-PME method. The production shock wave simulations were run with a small time step of 0.5 fs to better conserve energy throughout the numerical integration of the equations of motion, for a total of 100 ps. This way, shock waves with varying kinetic energies and corresponding amplitudes were generated to mimic ultrasound shockwaves.

In the experimental setup, the particles are dispersed throughout the liquid volume. The ultrasonic probe generates a high-intensity wave in its direct path, causing high turbulence and suspension mixing as a result. The particles in the direction right below the probe will receive the maximum possible energy from the sonic wave, while the other particles in the mixture receive less energy from the secondary propagation and mixing. As such, the simulations in a variety of kinetic energy shockwave conditions allow a comprehensive observation of the possible shocks according to both the US equipment intensity (power or amplitude) and the other possible secondary effects that do not happen in the direct path of the probe beam. The higher the intensity, the higher the energy of the shockwave hitting a particle, as well as how close the particle is to the path of the probe.

## Results and Discussion

### Flotation Tests

**Figure 2:**
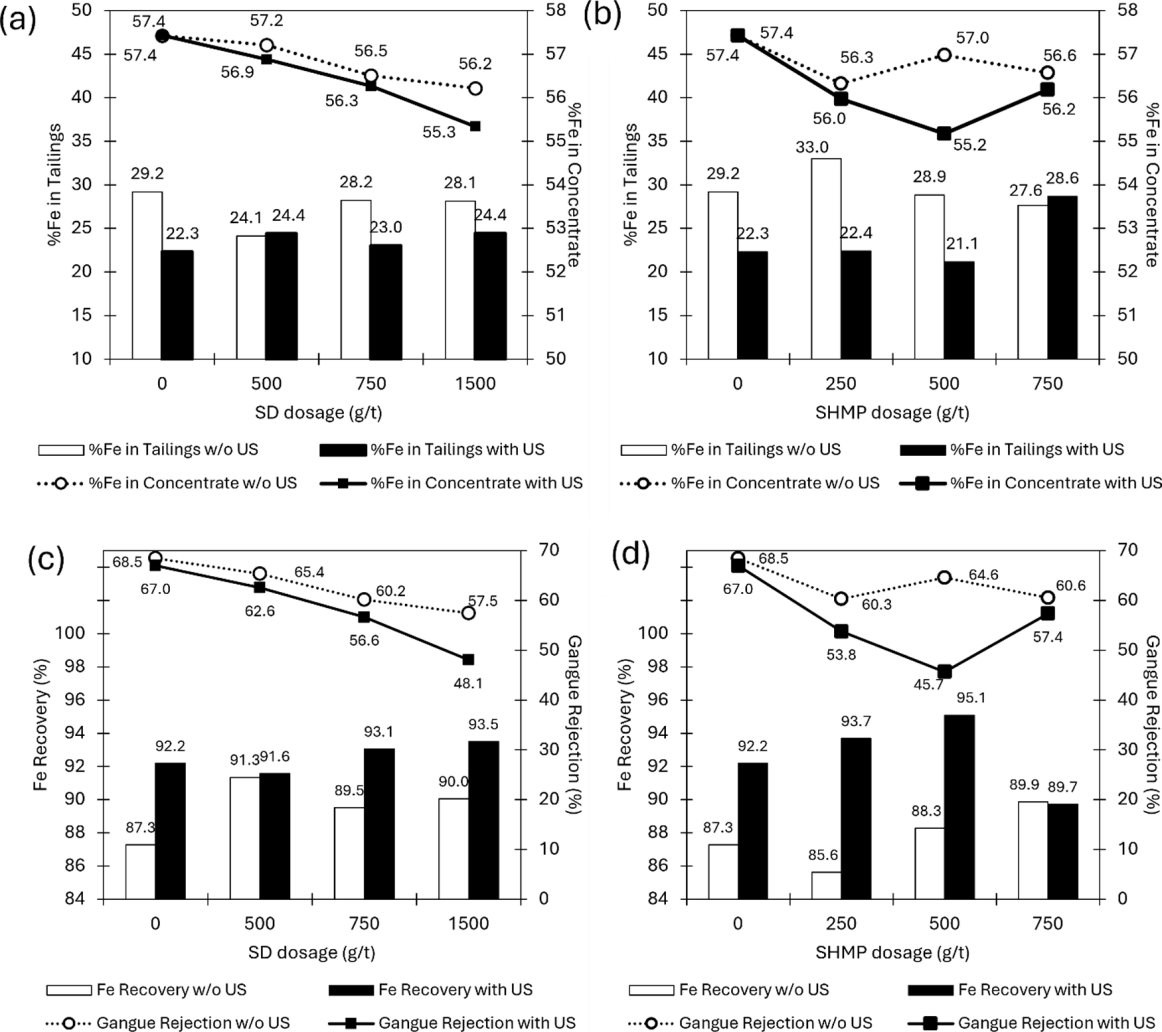
Flotation results with different dispersant dosages and ultrasound for SD (a,c) and SHMP (b,d). White and black bars and markers refer to conditions in the absence and presence of US, respectively.

Figure 2 shows the effect of chemical dispersion and ultrasound pretreatments on the flotation system. In general, the behavior observed with the application of dispersants is the reduction in %Fe loss to the tailings and enhanced metal recovery. Although the usage of the dispersant reagents was effective in lowering Fe loss by entrainment, it also depressed the gangue flotation, with a larger effect by increasing concentration. Previous reports have already pointed out no selectivity in using dispersant chemicals such as SHMP and they act as depressants for quartz and other minerals.^9−11^ It can be due to promoted particle–particle interactions or to the anionic dispersants jeopardizing the action of the cationic collectors. For example, by complexing in solution^9,52^ or making the surfaces too hydrophilic when adsorbing in high amounts, with hydration layers and counterions hindering the collector species approach.^11,44^

When applying US, Fe recovery was enhanced (up to 9.4%), and %Fe in tailings diminished (up to 10.6%), but the concentrate quality diminished too (minimum value 55% Fe), due to even higher gangue depression than in the absence of US. Both dispersants had lower performance under sonication, with their detrimental gangue depressing effect being enhanced, with the effect of US more pronounced in the presence of SHMP dispersant. The less significant increase in metallurgical recovery promoted by chemical dispersion alone, when compared with the application of ultrasound, can be explained by the additional strong mechanical forces and the detachment of slimes by cavitation promoted by ultrasound. This type of ultrafine particle with a high specific surface area demands strong mechanical forces to permit the effective action of the collectors subsequently. This has been reported before for similar ultrafine iron ore tailings using the same type of amidoamine collector.^12^ In that case, the effective separation was obtained only after proper high-intensity conditioning (HIC) prior to flotation. Similarly to US, HIC flotation enhancement mechanisms are attributed partially to the generation of smaller bubbles and particle surface cleaning, as well as beneficial aggregation between some of the particles.^12^ Additionally, the particle–particle interactions are strongly dependent on the agitation intensity (on the energy dissipation) according to Abrahamson's model when the fluid eddy’s size defined by Kolmogorov’s scale is comparable with particle size, thus promoting aggregation.^53^

### Sedimentation Tests

**Figure 3:**
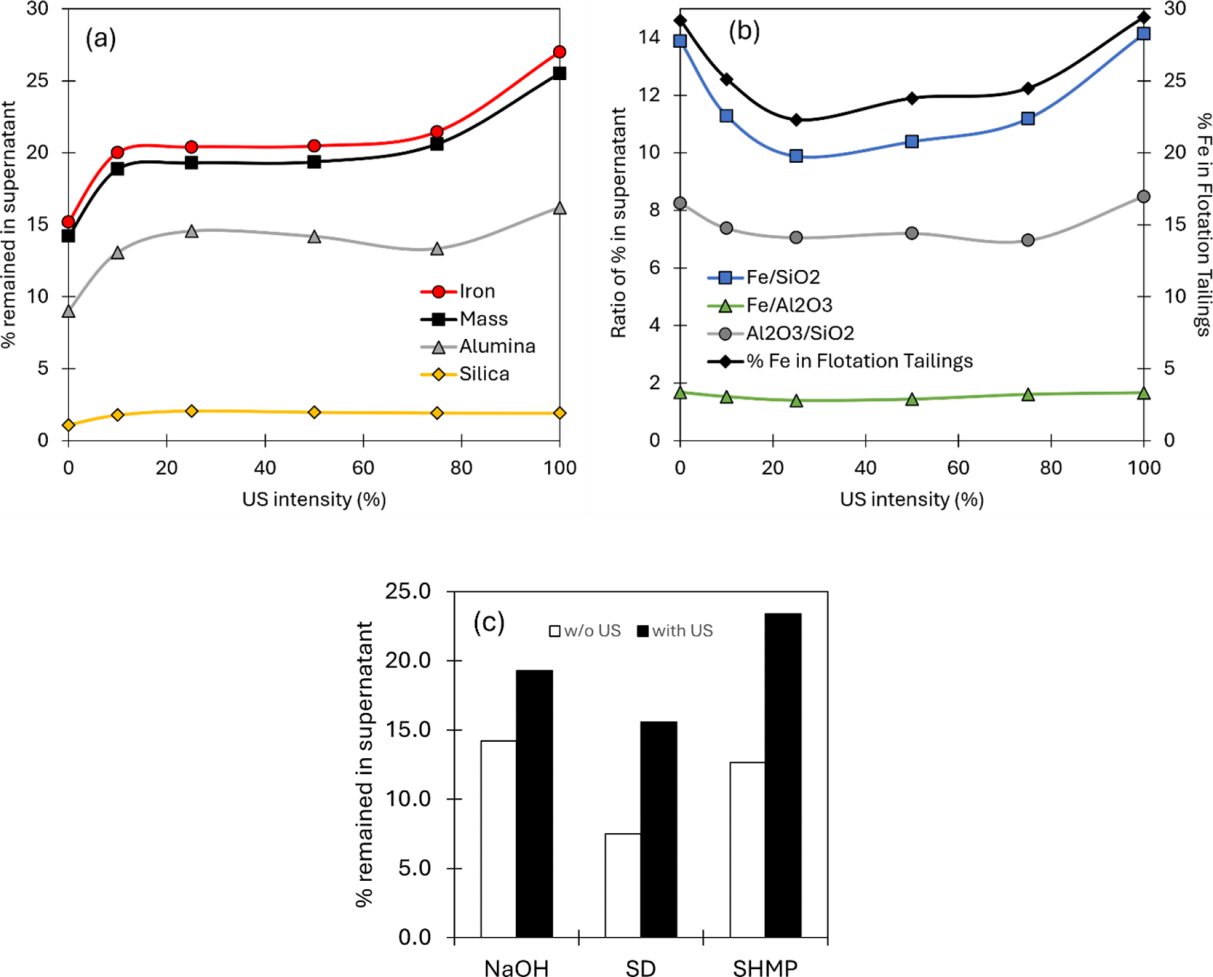
(a) Percentage of mineral species remaining in the sampled supernatant at a sample depth of 6 cm after 0.5 h and (b) ratios of the quantities in (a) compared to flotation %Fe in tailings. (c) Percentage of the mass that remained in the sampled supernatant in systems with chemical dispersants, SD at 750 g/t and SHMP at 500 g/t, and the effect of US at 25%.

**Figure 4:**
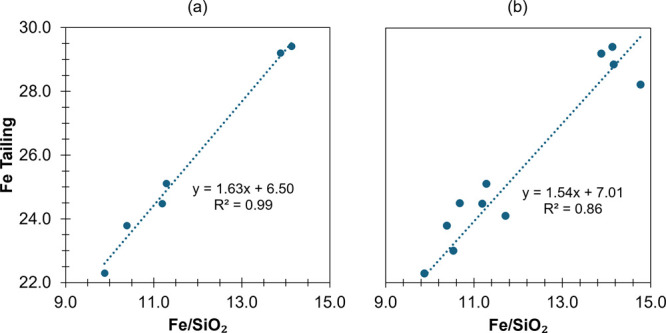
Correlations between flotation results for %Fe in the tailings in flotation and the Fe (%)/SiO_2_ (%) ratio in the supernatant phase (6 cm at 30 min) obtained from sedimentation tests. (a) Under US treatment with varying amplitudes (0–100%); (b) under US treatment with varying amplitudes and durations (5 and 15 min at 25%), and dispersion using NaOH, NaOH + US, SD, SD + US, SHMP, and SHMP + US.

To better understand the behavior of ore components during sedimentation under different US intensities, a series of sedimentation tests were conducted, as illustrated in Figure 3. Figure 3a displays the composition of the supernatant collected during these tests, showing the percentage of ore components that remained suspended as a function of the US intensity. As can be seen, from 10 to 75% US, the iron amount suspended does not change. For silica and alumina fractions, they both have a maximum at 25%. This indicates that at this sonication level, more silica and alumina fines are released in the pulp together. The alumina profile closely follows the iron profile and differs more from the silica profile, suggesting that the alumina-bearing slimes are more closely associated with the iron minerals, being also the two mineral components with higher percentage remained in suspension, while only a much smaller fraction of silica remains suspended. Using these values, we calculated the ratio of %Fe to %SiO_2_ and %Al_2_O_3_, which is a form of relative composition, revealing the data in Figure 3b. As can be seen, the shape of the curve resembles closely the shape of the %Fe in tailings from the flotation tests at the same US levels.^18^ This is ultimately shown in the linear correlations in Figure 4. The iron/alumina ratio in Figure 3b is almost constant, reinforcing the associated nature of the fine fraction of these two mineral groups. These results are in line with the previous investigations where the iron loss to tailings in this slime sample was determined to be majorly due to entrainment of the fines.^18^

Recalling the results of the referred previous work,^18^ where we compared the US effect under varying intensities and even compared the dispersion stage at natural and alkaline pH, the variation of US intensity showed that the flotation mass recovery, Fe recovery and %Fe in tailings had optimum values around 25% US intensity. There were only small variations between 20 and 75%, with a worse performance observed at 100% intensity. The results comparing dispersion pH variation and US showed that only at pH 10.5 plus US enhanced separation efficiency was reached. Particle size distributions pointed to the increase in the population of finer and coarser particles in comparison with the sample prior to the dispersion tests, indicating both slime coating separation and aggregation of another fraction of particles, which due to the good flotation selectivity, was attributed to possible homoaggregation of iron minerals.

In conditioning processes involving mechanical energy input such as agitation, mixing, high-intensity conditioning, and sonication, the hydrodynamic forces are responsible for the removal of slime coatings, which overcome the adhesion force and remove particles from surfaces. However, the high mechanical energy input exerts kinetic energy on particles and thus may improve collision and adhesion of fine particles too,^5^ and as such an excessive energy input is likely to increase slime coatings or other undesired aggregations, and thus the intensity of conditioning, and sonication in this case, is a key parameter that needs to be determined for this type of application. In this study, the 25% level was optimum.

Figure 3c shows the percentage of mass suspended according to the tests with the dispersants. In the absence of US, both SD and SHMP resulted in a smaller mass of dispersed particles. For both SD and SHMP, the use of US virtually doubled the mass suspended, almost reaching 25% for SHMP. The percentage of the mineral species follows the same trends, showing little selectivity.

**Figure 5:**
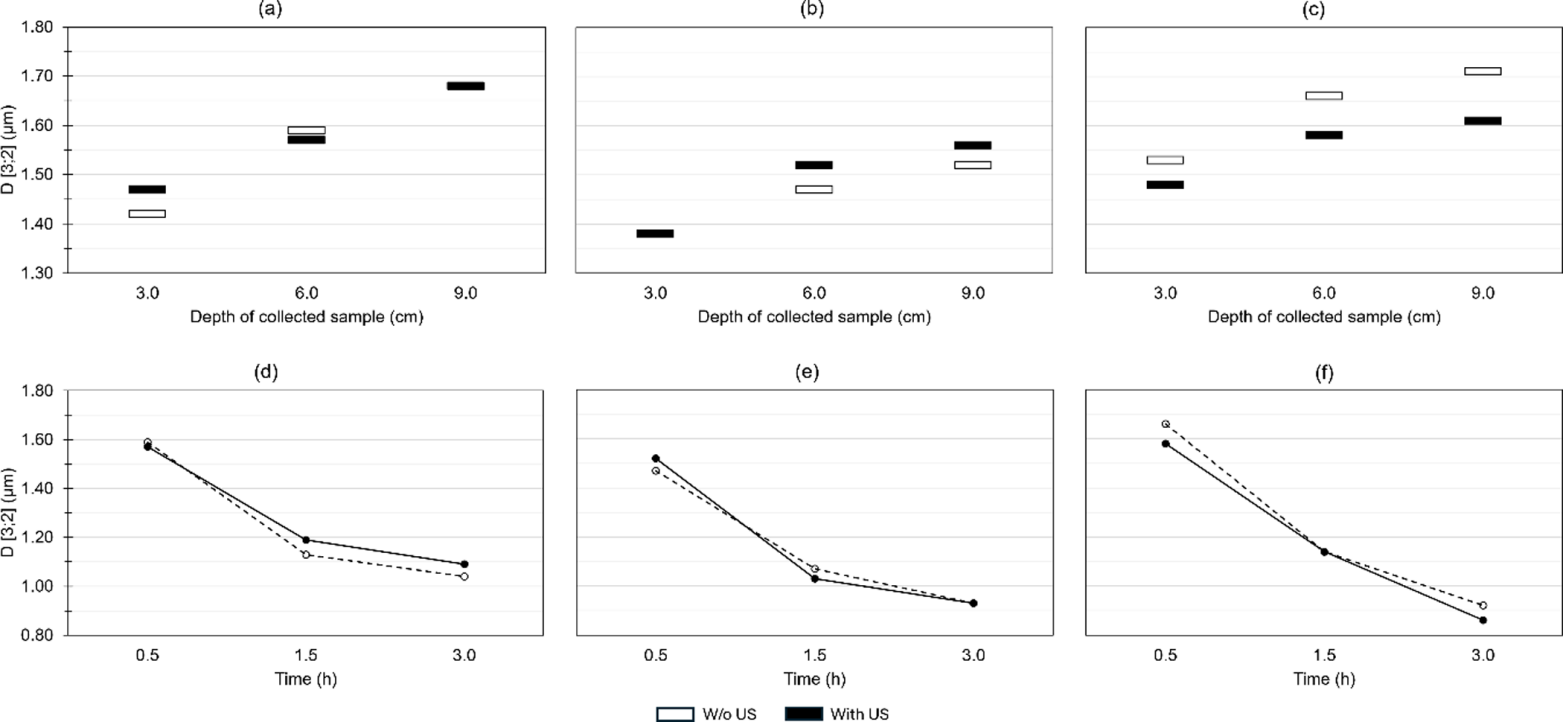
(a–c) Particle dispersion analysis and (d–f) sedimentation rate analysis from sedimentation tests for samples dispersed with (a,d) NaOH, (b,e) SD, and (c,f) SHMP, with and without US treatment.

Additionally, the effect of dispersants and US treatment on particle size distribution was analyzed using the Sauter mean diameter (D[3;2]) parameter. Without US treatment, particle dispersion analysis (Figure 5a–c) shows that SHMP is more effective at keeping slightly larger particles suspended compared to SD. Under US treatment, the average particle size increases in the presence of SD but decreases in the presence of SHMP.

The sedimentation rate analysis, obtained by extracting samples at different times (0.5, 1.5, and 3 h) from a fixed depth of 6 cm, shows only small differences between the behavior of the dispersants (Figure 5d–f). Sedimentation seems to be slightly faster with SHMP, as the decrease in particle size over time is more pronounced, as can be seen by the greater slope of the line. The effect of US on the sedimentation rate is also small, slowing particle sinking with NaOH and SHMP, and increasing it with SD during the first interval. In the second interval, this behavior reverses.

These results point out that the dispersion does not follow exactly the intended. The expected is that efficient dispersion will release more fine particles into the medium that will remain in the supernatant (higher mass % in the supernatant). In the base cases without US, the added chemicals caused less mass to be released to the supernatant. This indicates that, although they could have initially dispersed the particles, some aggregation must have been promoted, with the dispersants acting as coagulants and generating heavier particles that settled. These findings suggest that US pretreatment alone is a better process option than chemical dispersion, meeting the flotation results from Figure 2.

The dispersive chemicals used are sodium salts of polyanions. All minerals are negatively charged at the experiment’s pH of around 10,^33^ way above all mineral isoelectric points, especially quartz. As such, their adsorption must be favored by different driving forces rather than straightforward electrostatics. For the iron minerals, being less negative and possessing iron sites which are known to be easily complexed by phosphates, carboxylates, and other oxygen-bearing anionic groups, the explanation may be related to chemisorption.^7,54^ However, for quartz and kaolinite, the same is not true. Why then could the dispersants promote the silicates’ depression and possible aggregation? Some combined reasons can be hypothesized. As the dispersants are salts, not only are the active agents (the polyanions) present but also a high amount of sodium ions is introduced into the system. Recent reports have demonstrated that in such cases, the adsorption mechanism is promoted by salt bridges (or ion bridging), in which the Na^+^ cations adsorb onto the negative mineral surface while contacting the anionic groups of the adsorbing chemicals.^55−57^ Quezada et al. have demonstrated such a mechanism by detailed MD simulations of quartz and kaolinite surfaces under different pH (surface charge) and salinity conditions, considering polyacrylate oligomers, phosphate, and polyphosphate anions.^55−57^ The cations acting as bridges between two negatively charged groups, one from the mineral and one from the polyanion, drive the adsorption. In this case, the adsorption of these highly ionic and hydrophilic compounds could be enough to explain the depression mechanism, hindering the collector’s action. The number of ions involved can be enough to compress the electric double layer of the mineral particles and screen repulsive interactions, ultimately allowing them to aggregate by sharing an ionosphere, in a colloidal version of the ion bridge phenomenon,^58−60^ which has been demonstrated in recent works, proving the aggregation of like-charged particles. The use of US combined with the dispersants, worsening overall flotation, can be possibly related to the extra high energy input, helping in the adsorption of the dispersants to extents that further increase the depressing effect, and also helping particles surpass the energetic barrier of long-range electrostatic repulsion, facilitating collision and contact, which is then maintained by the sharing of counterions.^58−60^ That aligns with the same strategy used to create the slime-coating models in this work.

### Equilibrium MD Simulations

The initial equilibrium simulations showed the expected repulsion between the like-charged particle and surface, quickly establishing a separation distance, while counterions solvated them. These configurations remained stable for 80 ns (Figure 1b,e), which is the expected behavior in response to pH and chemical dispersion effects. However, for particles that are strongly adhered, separation is more difficult, because the contact surfaces are not easily accessible to the solution and require some external force, typically provided by mechanical agitation.

The first set of SMD simulations showed that, even though the particle and surface pairs were like-charged and repelled each other if enough energy was applied, they could overcome the energetic barrier and aggregate through shared interfacial counterions by ion bridging^58−60^ (Figure 1c,f). These systems remained stable for 90 ns and were then analyzed under equilibrium conditions. This demonstrates that even when dispersant chemicals (that should enhance electrostatic repulsion) are used, high energy input, such as in sonication, can favor particle aggregation. This effect is further discussed in the experimental section, where the antagonistic effect of ultrasound with dispersants on flotation results is examined.

To replicate extreme sonication effects, we simulated goethite over quartz under varying temperature and pressure conditions. The systems remained highly stable at 298, 353, and 500 K and showed no separation. In fact, closer contact and aggregate stabilization were observed. At higher temperatures of 750 and 1000 K, rapid water vaporization led to a particle-surface separation. However, when simulating at 1000 K and 1000 bar or under constant volume (ambient water density), the system remained adhered, with further stabilization and closer contact. These results indicate that temperature alone does not effectively promote particle separation. Instead, higher temperatures increase the water mobility, which aids in adjusting the particle-surface configuration and enhances adhesion. As long as water remains in the liquid phase, an increase in temperature favors particle aggregation by enhancing water diffusion, reducing viscosity, and lowering the energy barrier posed by the strong interfacial hydration layers. When vaporization occurs, expansion of the gas phase can separate the slime coating from the surface.

These findings align with experimental results, where metal recovery, mass recovery, and iron % in tailings did not correlate well with pulp temperature, and gangue rejection and iron % in concentrate showed only little tendency to lower with temperature (and US intensity), all with low correlation coefficients (see Figure S1 in the Supporting Information).

### Nonequilibrium MD Simulations

**Figure 6:**
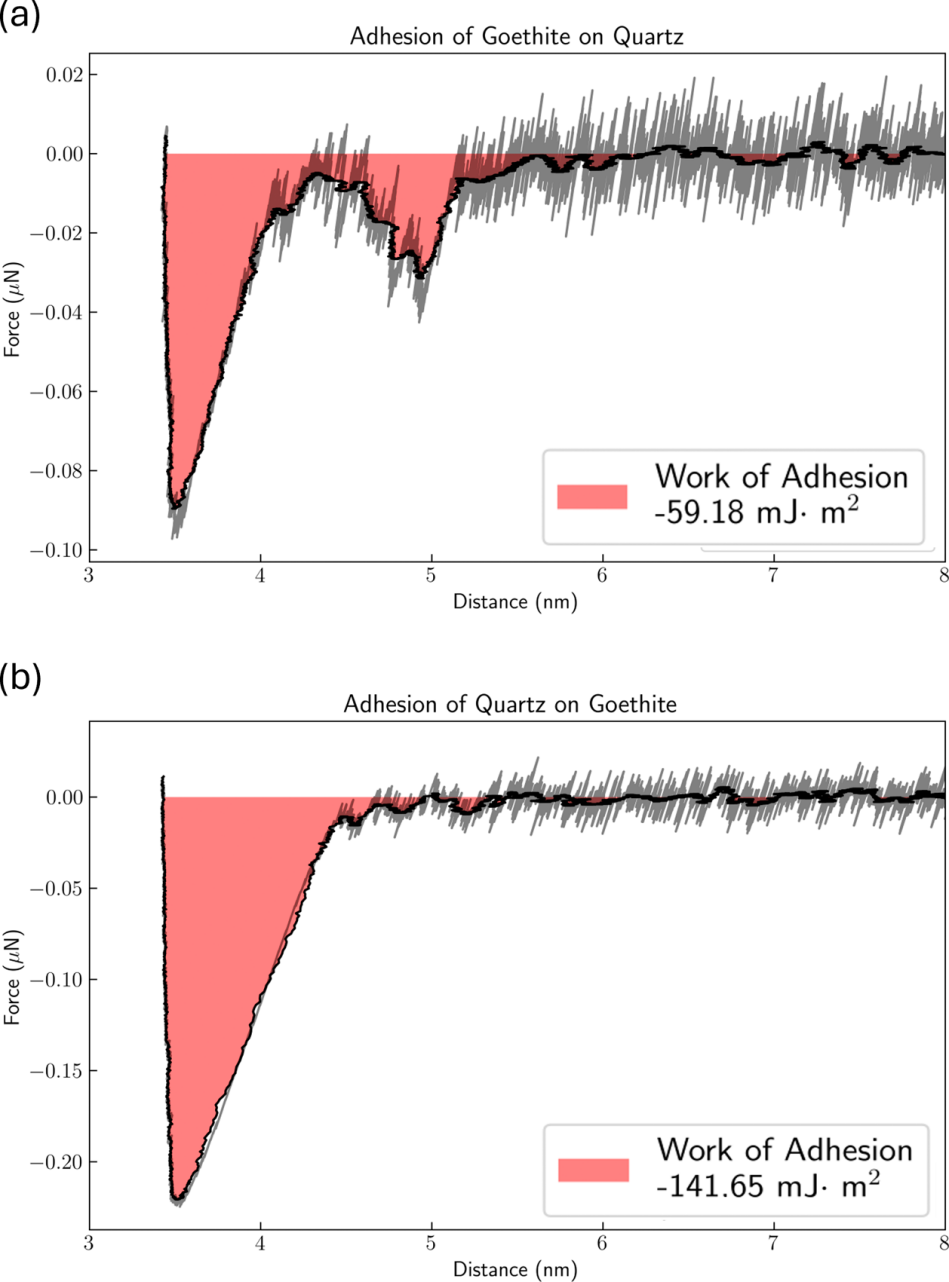
Force versus distance curves for the detachment of the slime coating models via SMD and the respective work of adhesion for (a) goethite particle onto the quartz surface and (b) quartz particle onto the goethite surface.

After equilibrium simulations, SMD was used to pull the particle into the solution to measure the corresponding force curves and detachment work (Figure 6), which indicate stronger adhesion of the quartz particle to the goethite surface rather than the other configuration, most possibly due to the more negative surface charge of the quartz particle and the more structured hydration of goethite.^44,61^ Goethite and other metal oxides can form inner-sphere complexes with Na^+^ through their hydroxyl oxygens, resulting in more specific and stronger interaction energies than those of quartz when the surfaces are neutral.^44,61^ In that sense, when pulling the goethite particle from the quartz surface, the inner-sphere goethite-bound Na^+^ cations are kept together with their preferred sites, while when pulling the quartz particle from the goethite surface, the higher negative charge of quartz tries to pull the Na^+^ cations together from their sites in the goethite surface. The magnitude of quartz particle adhesion is 2.4 times that of the goethite particle for these model systems, with 141.65 versus 59.18 mJ·m^–2^, or in native simulation units, with 2131.9 kJ·mol^–1^ versus 890.7 kJ·mol^–1^. These values of work of adhesion are in the range of observed experimental values and serve as a guide for understanding the effect of the sonic waves.^62−64^

### Shockwave Simulations

**Figure 7:**
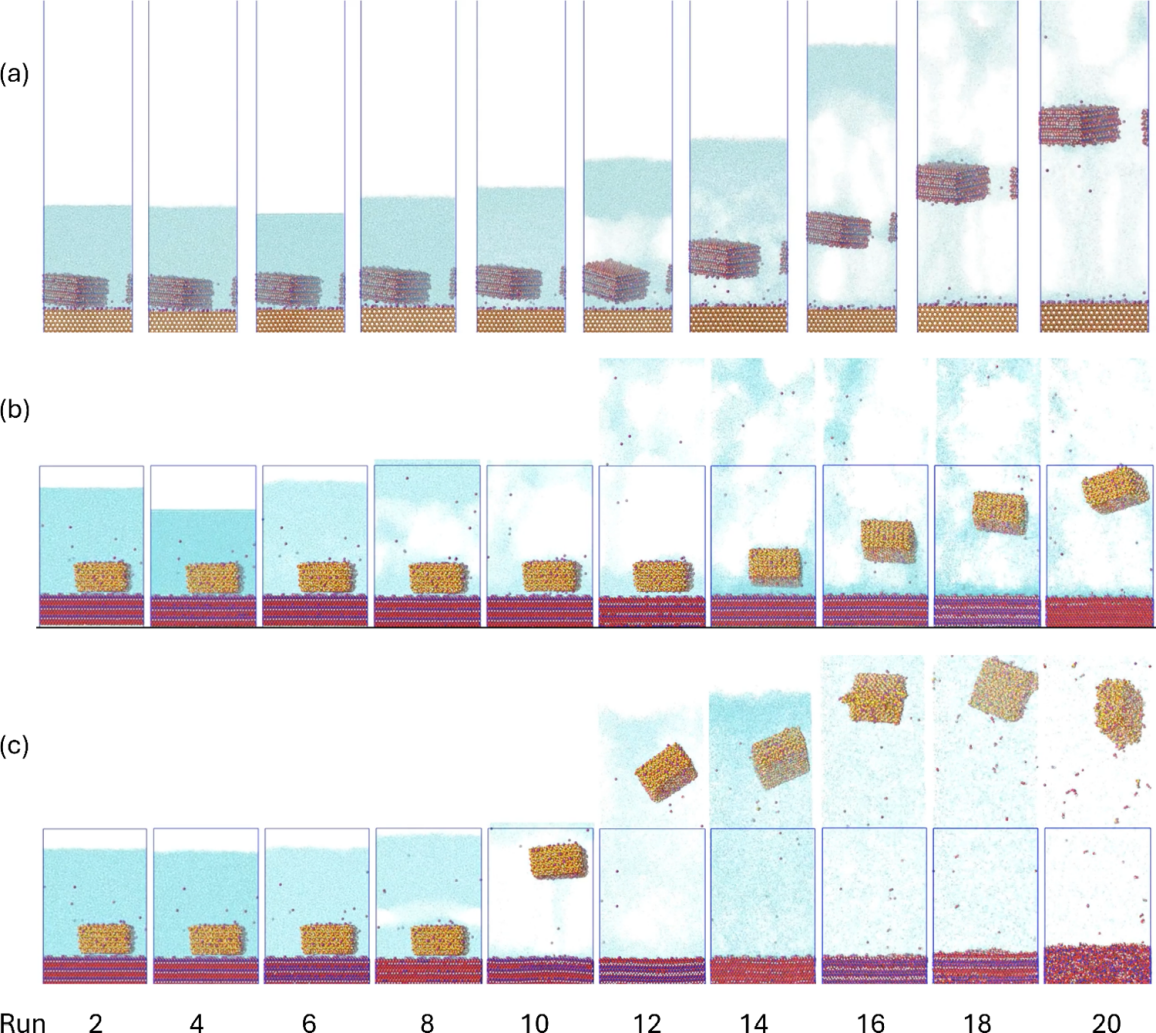
Representative snapshots from the shockwave simulations. (a) G-on-Q after primary shock; (b) Q-on-G after primary shock; and (c) Q-on-G after secondary shock.

The implemented protocol enabled observation of the effect of the shockwave intensity on particle-surface aggregates. Figure 7 presents representative snapshots from the simulation trajectories at different initial input kinetic energy. We encourage the reader to watch the simulation videos provided as Supporting Information, as they offer a more detailed illustration of the simulations and complement the descriptions in the main text. Table 4 summarizes the parameters used in these simulations. After preliminary tests, we defined initial velocities that would allow observation of a range of behaviors. Since the *W*_a_ measured from SMD was almost 3 times larger for the Q-on-G system, the range of velocities and energies were designed to be close to 3 times larger than for G-on-Q too.

**Table 4:** Input Kinetic Energies and Velocities of Shockwave Systems

	goethite particle onto the quartz surface (G-on-Q)		quartz particle onto the goethite surface (Q-on-G)	
system run	added kinetic energy (kJ mol^–1^)	*v_z_* (nm ps^–1^)	added kinetic energy (kJ mol^–1^)	*v_z_* (nm ps^–1^)
1	8407.9	0.11	22066.9	0.16
2	20835.3	0.17	68728.9	0.27
3	42485.4	0.25	139161.4	0.39
4	74273.7	0.33	241296.5	0.51
5	114790.4	0.41	375269.6	0.64
6	164402.7	0.49	536433.1	0.76
7	223710.5	0.57	726737.1	0.89
8	291681.3	0.65	947189.4	1.02
9	369320.0	0.73	1196244.2	1.14
10	455330.0	0.81	1474959.2	1.27
11	550898.7	0.90	1782175.1	1.39
12	656002.4	0.98	2119795.9	1.52
13	769981.2	1.06	2486025.9	1.65
14	893402.6	1.14	2881547.0	1.77
15	1026026.6	1.22	3307121.2	1.90
16	1166959.5	1.30	3761914.0	2.03
17	1317739.5	1.38	4245820.8	2.15
18	1477655.2	1.47	4758943.0	2.28
19	1646025.2	1.55	5301676.3	2.40
20	1824687.5	1.63	5873545.5	2.53

**Figure 8:**
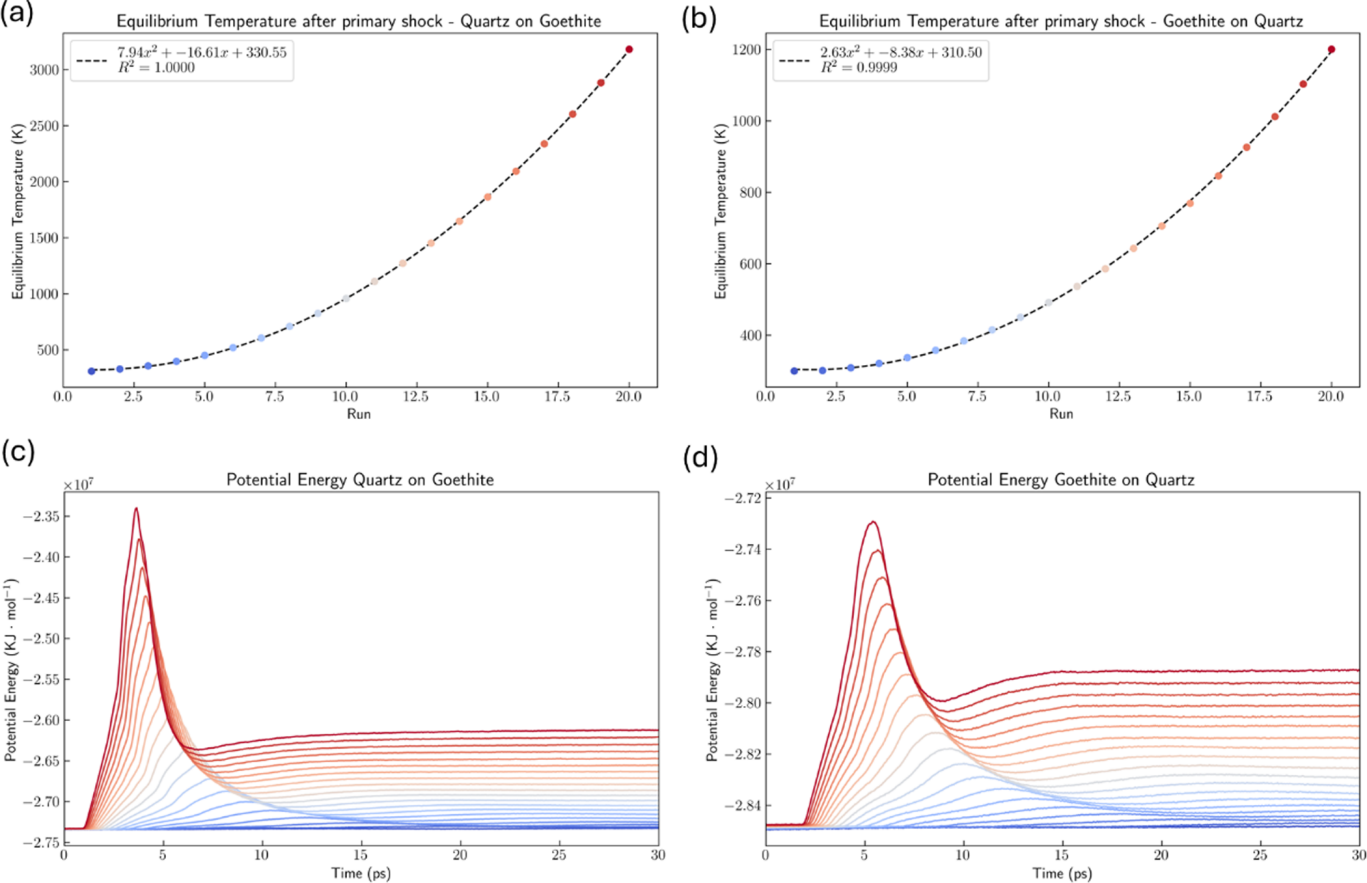
Temperature rise after primary shock for: (a) quartz particle onto goethite surface; (b) goethite particle onto quartz surface. Potential energy x time for (c) quartz particle onto goethite surface and (d) goethite particle onto quartz surface.

As seen in Figure 8a,b, after the shocks, the system temperature rises significantly due to the dissipation of kinetic energy through the surrounding molecules, reaching localized temperatures of several thousand Kelvins, as reported in the literature.^14^Figure 8c and d illustrate the potential energy of the systems, starting from the same initial level and then reaching different values according to the energy change, converting the input kinetic energy into potential energy and reaching a maximum in the peak region, equivalent to the minimum distance in the compression regime.

**Figure 9:**
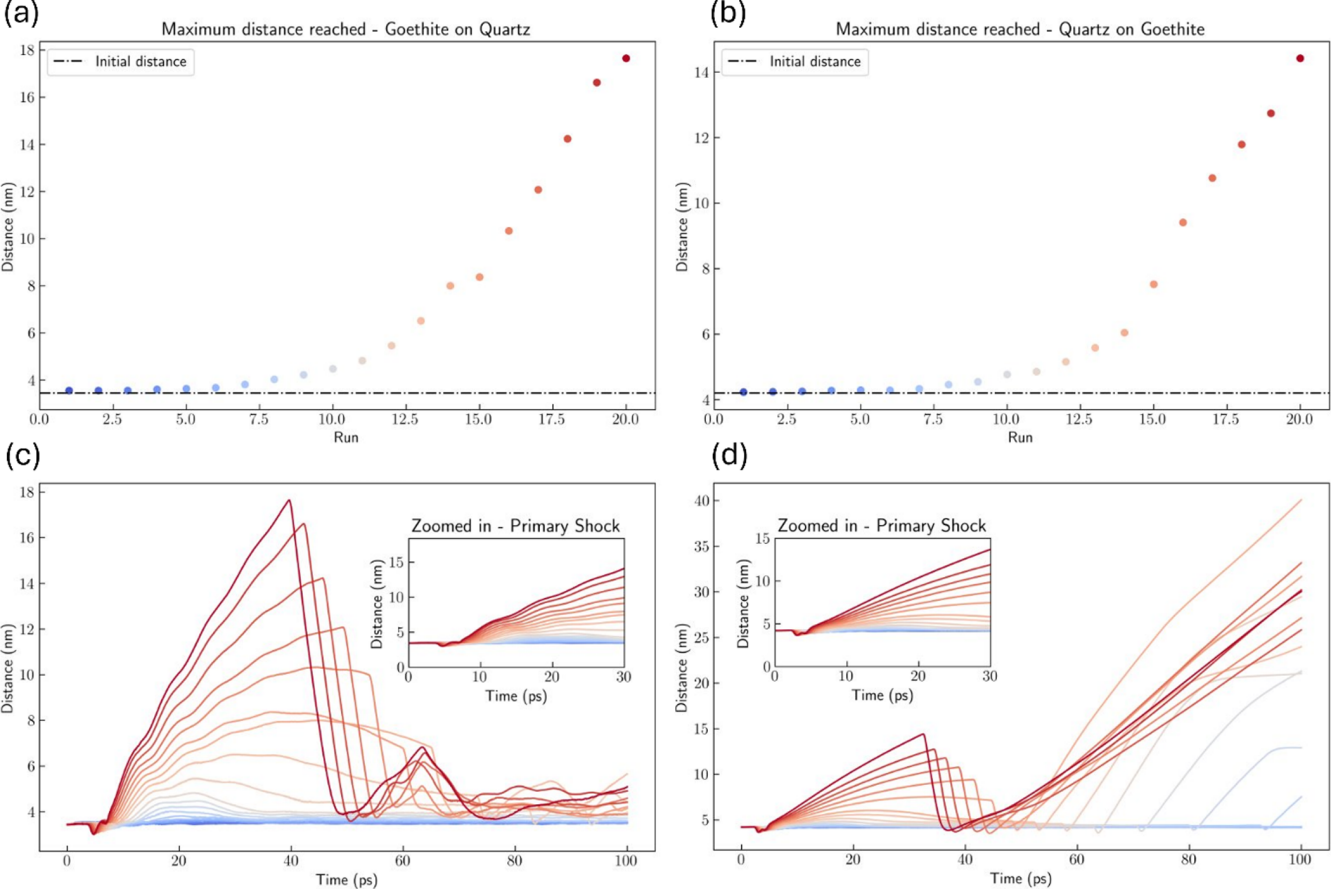
(a,b) Maximum particle-surface distance achieved after the primary shock. (c,d) Particle-surface distance as a function of the simulation time. Left column: G-on-Q systems; Right column: Q-on-G systems.

Figure 9a,b shows the maximum separation distance between the particle and the surface after the primary shock. As expected, the particle detachment phenomena were a function of the amount of input energy, with the systems at low input conditions displaying little effect on the aggregate separation. After a threshold, an initial detachment can be achieved (Runs 6 onward). However, in most cases, particles reattach quickly but at a slightly higher distance as can be seen in Figure 9c,d, which display the separation distance along the trajectory. Increasing the shock energy facilitates easier separations at first, and when the shock is strong enough, we observe rapid detachment with increasing separation distances. However, with even higher energy, the systems collide with the opposite boundary wall, causing a secondary shock, that can drive particle and surface to collide again. Correlating to the experimental physics, these secondary shocks in the simulations are equivalent to successive sonic waves or the collision with other particles in the suspension as promoted by the intense mixing.

Cavitation phenomena were observed at energy inputs lower than those required for particle detachment. The formation and subsequent collapse of bubbles play a crucial role in ultrasound energy transmission and the micromixing effect. Although the input kinetic energies were higher than the needed amount as probed from the SMD simulations, the waves hit the particle-surface systems in the normal direction, initially forcing the particle closer to the surface (positive pressure, compression regime) and then expanding back (negative pressure, rarefaction regime), when the energy more effectively affects water. As such, causing heating and cavitation before detachment can occur. As the energy input increases and cavitation becomes faster and more intense, detachment is observed. When the energy input is even higher, vapor keeps expanding and the systems gain velocity in the opposite direction, ultimately suffering a secondary shock when it hits the other boundary wall. These secondary shocks force the bubbles to collapse, causing vigorous turbulence. The particles are projected against the surface, reattaching in some cases (high energy G-on-Q systems) or when energy input is too high, causing breakage and deformation, as well as repelling again (high energy Q-on-G systems). Depending on the energy input, the secondary shockwaves promote the reattachment, reinforcing the previously mentioned effects of high energy mixing, and promoting aggregation.

Our results show that quartz slime coating onto goethite is more strongly bound than goethite slime coating onto quartz. Only from Run 15 onward (added kinetic energy = 3,307,121.2 kJ·mol^–1^, *v_z_* = 1.90 nm·ps^–1^), did the primary shockwave promote the quartz particle stable detachment, while for the goethite particle, this was attained at Run 14 (added kinetic energy = 893,402.6 kJ·mol^–1^, *v_z_* = 1.14 nm·ps^–1^). This difference is ultimately related to their surface charge density under the studied pH condition. This is in line with the SMD pulling simulation results. The values of the needed input kinetic energy to stable detachment are approximately 1000 times larger than *W*_a_. As previously said, this energy input is transmitted (and dissipated) through the whole system, so only a fraction of it goes for the specific “task” of separating the particle from the surface, while in the SMD approach, the energy measured is directly applied to that task.

The simulations of quartz slime coating under high shock energy revealed not only particle separation but also the breakage of surface groups. Despite the approximations inherent in the models, this provides qualitative evidence of the chemical effects of high-energy ultrasound, which has been reported in the literature to cause surface alterations, bond breakage, and even radical formation.

This relationship between wave intensity and separation efficiency highlights the sensitivity of the process to specific conditions, emphasizing the importance of optimizing particle separation. As demonstrated, ultrasonication plays a clear role in enhancing dispersion, which alone can be a paramount factor for enhancing the efficiency of sequential flotation separation or other processes that benefit from chemical-free particle dispersion. However, other effects could help to enhance the efficiency. As indicated by our simulations, the shockwaves can cause strong surface modifications. Breakage, fissuring, and cleaning processes may all happen, leading to alterations of exposed surface groups and higher surface roughness. Such effects combined may also cause enhancement of some reagent-surface interactions as well as hinder others. These effects have been reported in the literature to influence the flotation response.^65,66^ Here, our main objective was to obtain a molecular picture of the sonication-induced subprocesses instead of a quantitative description. Nevertheless, these are the first steps to deepen our understanding of these systems toward predictive quantitative modeling in future works.

### Stable Nanobubble Cavitation and Particle Damage Microprocesses

**Figure 10:**
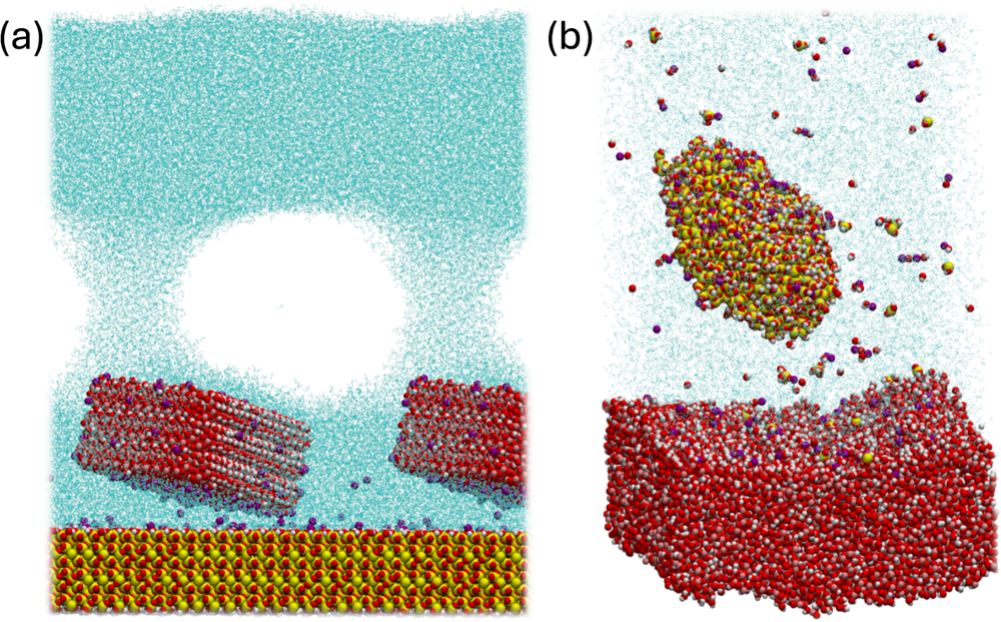
Emphasis on relevant microprocesses in US systems: (a) Stable nanobubble formation and (b) surface breakage and deformation effects.

Emphasis must be given to some relevant microprocesses observed in these simulations. It is known how the US promotes cavitation. Although all simulations run at energy levels high enough to lead to the cavitation phenomena, with water vaporization, in a few cases, we observed the formation of stable nanobubbles. Run G-on-Q number 12 is a perfect example. In this scenario, the energy input was enough to promote cavitation but not too high to promote increasing vapor expansion (Figure 10a). It is known that stable bubbles can be further collapsed by the follow-up of another pressure wave, creating high-speed jets that can damage surfaces and disrupt particle aggregates.^5^ Also, stable nano and microbubbles may remain in the suspension in the bulk and adhere to particles, facilitating subsequent flotation processes.^17,67,68^ That has been an increasingly relevant topic in mineral processing and water treatment fields.^69,70^ Ultrasound is not the only method used to create nanobubbles but is one of the most widespread. The nanobubbles can serve as nuclei for enhanced adhesion of micro- and/or macrobubbles, acting as collectors. They have also been shown to allow for the aggregation of particles in the pulp. In some flotation processes, this has been attributed as the mechanism for enhancing fine particle flotation, with the nanobubbles forming flocs with the fine particles, becoming an apparent bigger but lighter particle, easier to be carried by regular bubbles, and with enhanced kinetics.

Another significant effect of the US is surface breakage and damage. At sufficiently high energy inputs, the sonic waves can destroy particle surface structures. The high-energy Q-on-G runs are examples. In these cases, we observed the breakage of surface structures of the quartz particles, resulting in protrusions and detachment of silicate groups. At the highest energy input levels, the goethite surface also suffered considerable damage, disorganizing its crystal structure after the secondary shock (Figure 10b). Breakage and surface modification effects have been reported by Gungoren et al. when studying quartz under ultrasound.^65^ They observed an increase in surface roughness and a decrease in particle size after US treatment (20 kHz and 30 W). They also obtained a similar level of pulp heating as we did and highlighted the importance of this effect, which should be controlled so as not to overheat the system and cause negative effects. Tang et al. studied the effect of ultrasound on the dissolution enhancement of several minerals related to surface alteration, bond rupture, and atomic dislocation.^22^ Others have reported similar observations on surface property alterations.^20^ These aspects can be explored to enhance selective collector adsorption, for example.

## Conclusions

In this work, chemical dispersion and ultrasonication were compared as flotation pretreatments for the reclamation of iron values from goethite-rich slime tailings. The results show that ultrasonication is more effective than chemical dispersants in achieving better separation. While dispersants can promote dispersion, only ultrasonication significantly enhances metal recovery without compromising the concentrate quality. Metallurgical recovery increased by up to 9.4%, with a concentrate quality loss of around 2 percentage points compared to less than 5% recovery improvement with chemical dispersion alone. The combined use of dispersant reagents and sonication displayed antagonistic behavior. The analyses from sedimentation tests correlated well with the flotation performance. For practical aspects, ultrasonication at moderate intensities is more efficient than chemical dispersion and does not contaminate the system with more chemical species, which can cause other detrimental effects.

We successfully applied molecular dynamics simulation protocols to study the effect of sonic shockwaves on the separation of mineral colloidal aggregates for the first time in the literature using representative slime coating models regarding iron ore processing. Our experimental results pointed out the positive effects of applying ultrasonication for dispersing the fine particles (up to a level of energy input) in comparison to traditional pH raising with NaOH or conditioning with dispersants such as SHMP alongside strong mechanical stirring. The nanoscale analyses showed how the imposed energy from the sound waves can cause vigorous turbulence associated with the local temperature increase, vaporizing the liquid water and separating the strongly bound particle-surface pairs. We observed bubble formation and collapse phenomena due to sonication-induced cavitation, as well as surface breakage and damage effects, widely reported in the literature. There is a threshold energy input to allow for stable particle detachment. Below it, particles barely separate or reaggregate. If too much energy is input, the particle–particle collision probability is raised, causing reaggregation. These results qualitatively describe the experimental behavior, for which, at higher sonication intensities or times, worse flotation efficiency was obtained. A molecular picture was established, providing valuable insights as the first steps for model refinements toward quantitative predictions. An in-depth analysis of the energy thresholds, in both simulations and experiments and the proper connection between them are relevant topics for future studies.

With this work, we advance the fundamental comprehension of microscopic phenomena happening in these mineral colloidal systems. We presented the usage of an unprecedented methodology to study the effect of sonic shockwaves on these types of systems by large-scale, state-of-the-art nonequilibrium molecular dynamics simulations. Our results pave the way for a higher understanding of sonication effects at the molecular level in particle dispersion as well as its implications for industrially relevant synthesis, separation, and concentration processes.

## Supporting Information Available

Correlation graphs between ultrasound generated pulp temperature and %Fe in concentrate, %Fe in tailings, metal recovery, mass recovery, and gangue rejection in flotation (PDF)
Video of initial repulsion (AVI)
Video of the G-on-Q simulation (AVI)
Video of the Q-on-G simulation (AVI)

## Supplementary Material

la4c03676_si_001.pdf

la4c03676_si_002.avi

la4c03676_si_003.avi

la4c03676_si_004.avi
