# Supplementary material for: Ultrasonic Dispersion for Iron Recovery from Slime Tailings: Microprocesses Unveiled through Molecular Dynamics Simulations
Source: Langmuir. 2025 Mar 17;41(11):7235–50. doi: 10.1021/acs.langmuir.4c03676 (PMC11948480; doi:10.1021/acs.langmuir.4c03676)
Supplement: Supplementary file 1 — la4c03676_si_001.pdf [file la4c03676_si_001.pdf]

Supporting Information for

Ultrasonic Dispersion for Iron Recovery from Slime Tailings: Microprocesses Unveiled Through Molecular Dynamics Simulations

Lucas Andrade Silva [1], Letícia Maia Prates [1*], Alexandre Moni Pereira [1], Julio Cesar Guedes Correia [1], Michelle Lacerda Sales Marques [2,3], Inna V. Filippova [3], Lev O. Filippov [3]**

[1] Centre for Mineral Technology (CETEM) – Ministry of Science, Technology and Innovation (MCTI), Molecular Modeling Laboratory, Av. Pedro Calmon, 900, Ilha da Cidade Universitária, Rio de Janeiro, RJ, 21941-908, Brazil

[2] Beneficiation Development Team, Belo Horizonte, Minas Gerais, 34006-270, Brazil

[3] Université de Lorraine, CNRS, GeoRessources, F54000 Nancy, France

* Corresponding authors: lucasandrade.chem@gmail.com ; lmprates@cetem.gov.br ; lev.filippov@univ-lorraine.fr

Table of Contents

Figure S1 – Correlation between ultrasound generated pulp temperature and (a) %Fe in Concentrate, (b) %Fe in Tailings, (c) Metal Recovery, (d) Mass Recovery, (e) Gangue Rejection in flotation……………………………………..S2

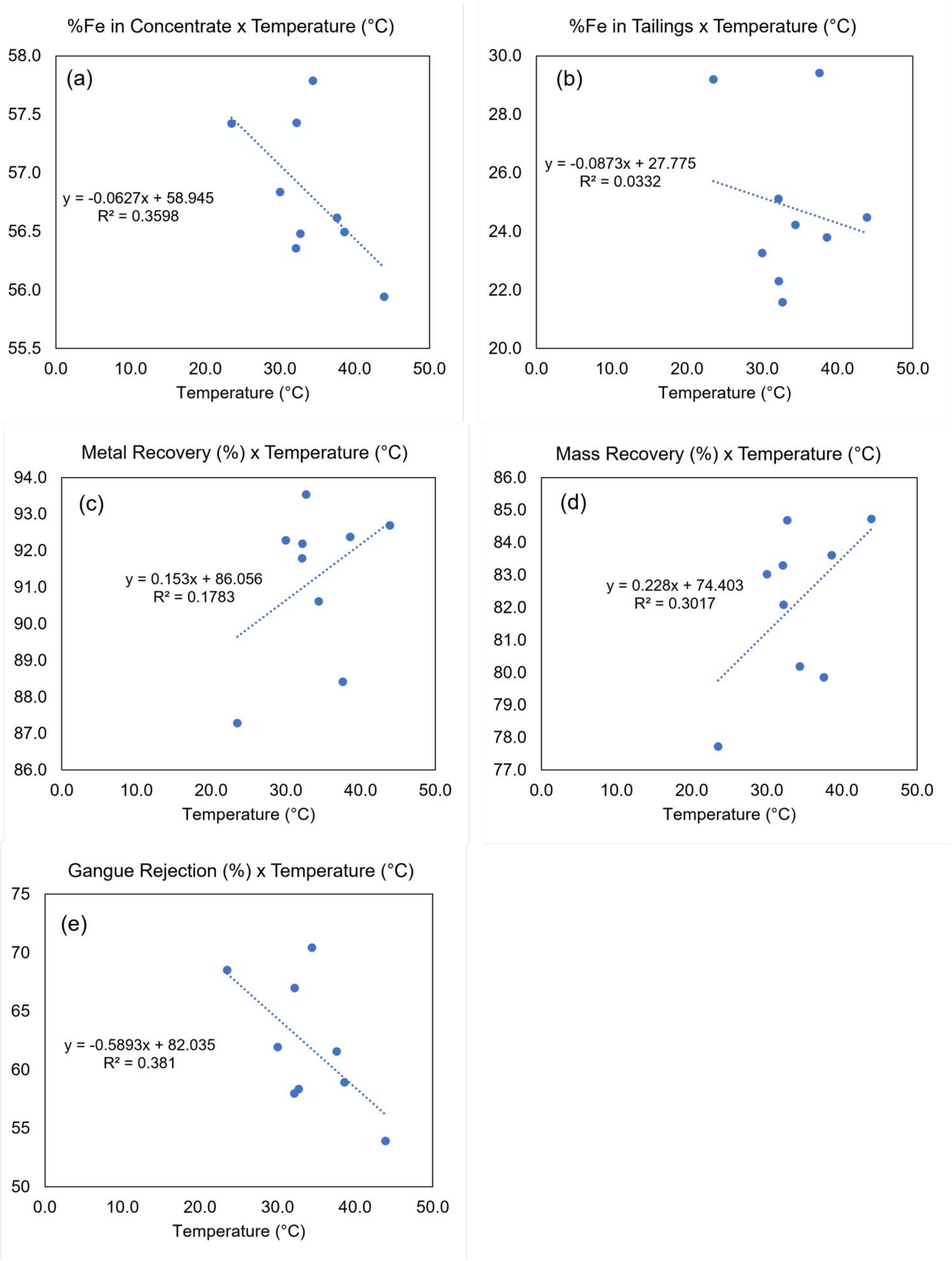

Figure S1 – Correlation between ultrasound generated pulp temperature and (a) %Fe in Concentrate, (b) %Fe in Tailings, (c) Metal Recovery, (d) Mass Recovery, (e) Gangue Rejection in flotation.
